# Collective Learning and Optimal Consensus Decisions in Social Animal Groups

**DOI:** 10.1371/journal.pcbi.1003762

**Published:** 2014-08-07

**Authors:** Albert B. Kao, Noam Miller, Colin Torney, Andrew Hartnett, Iain D. Couzin

**Affiliations:** 1 Department of Ecology and Evolutionary Biology, Princeton University, Princeton, New Jersey, United States of America; 2 Centre for Mathematics and the Environment, University of Exeter, Cornwall, United Kingdom; University of Chicago, United States of America

## Abstract

Learning has been studied extensively in the context of isolated individuals. However, many organisms are social and consequently make decisions both individually and as part of a collective. Reaching consensus necessarily means that a single option is chosen by the group, even when there are dissenting opinions. This decision-making process decouples the otherwise direct relationship between animals' preferences and their experiences (the outcomes of decisions). Instead, because an individual's learned preferences influence what others experience, and therefore learn about, collective decisions couple the learning processes between social organisms. This introduces a new, and previously unexplored, dynamical relationship between preference, action, experience and learning. Here we model collective learning within animal groups that make consensus decisions. We reveal how learning as part of a collective results in behavior that is fundamentally different from that learned in isolation, allowing grouping organisms to spontaneously (and indirectly) detect correlations between group members' observations of environmental cues, adjust strategy as a function of changing group size (even if that group size is not known to the individual), and achieve a decision accuracy that is very close to that which is provably optimal, regardless of environmental contingencies. Because these properties make minimal cognitive demands on individuals, collective learning, and the capabilities it affords, may be widespread among group-living organisms. Our work emphasizes the importance and need for theoretical and experimental work that considers the mechanism and consequences of learning in a social context.

## Author Summary

Learning is ubiquitous among animal species, allowing individuals to adjust their behavior in response to their environment to improve their chances of survival and reproduction. However, while many animals live and make decisions within social groups, it is not well understood how associative learning functions within a social context. We describe an empirically derived model of collective learning and compare the learned performance of animals within groups to the optimal behavior for a wide range of environmental conditions and group sizes. We find that the learning rules derived from experiments with individual animals readily generalize to a social context, and these relatively simple rules result in behavior that is close to optimal, even when individuals know neither the size of their group nor the properties of environmental cues. Individuals that learn in isolation and subsequently join together as a group make substantially worse decisions. These results demonstrate the importance of learning within a collective context and highlight the need for experimental work to investigate the role of collective learning in enhancing decision accuracy in animal groups.

## Introduction

Associative learning tunes an organism's behavior to exploit statistical patterns in the environment and can improve decision-making accuracy across a wide range of scenarios [1]–[2]. In the vast majority of experiments on learning, the subject of study has been a single individual in isolation (see [3]–[4] for reviews). When learning alone, there is a direct relationship between an animal's intentions and its actions: the animal observes cues in the environment and performs a behavioral response. The consequences of the behavior (such as a reward or punishment) may alter the animal's valuation of the environmental cues, resulting in a feedback loop that gradually tunes its behavior to its environment [3]–[7].

In contrast to this relatively simple scenario, many animals – including the majority of species commonly employed in learning experiments, such as rats, pigeons, and humans – live and forage naturally in social groups. Sociality offers many benefits to individuals, including improved sensing and decision-making [8]–[9], decreased risk of predation [10]–[16], improved foraging success [8], [16]–[21], and the capacity for thermoregulation [22]. For these and other species (e.g., fish [8], [16]–[18], [20]–[21], [23], birds [24]–[25], ants [20], honeybees [26], cockroaches [27] primates [28]–[29], and meerkats [30]), decisions are not made in isolation. Instead, in order to preserve the benefits of sociality, animal groups often must come to a consensus regarding where and when to travel or forage, despite the presence of dissenting opinions. While not universal amongst social animals, consensus decision-making is widespread in nature [15], [31]–[34], but does not necessarily imply that individuals are altruistic or highly cooperative. While members of some groups may be highly related (such as ants, honeybees, and primates), for many other species (such as some fish and birds), group members are unrelated to each other, and individuals obtain direct fitness benefits from maintaining group cohesion. These benefits provide a strong incentive for individuals to remain together, providing a platform for other emergent phenomena such as collective learning, which we explore here.

A common means by which consensus is achieved in animal groups is through relatively local responses to the positions or motion of others. Thus, in many species, such as schooling fish [16], [23], [35]–[37] or flocking birds [25], [38]–[39], individuals must reconcile any personal directional preferences with their social tendency (to avoid isolation, and to copy the movement decisions made by others). Spatially explicit models of collective movement (or ‘swarming’) are commonly employed to describe mobile animal groups. In these models, individuals interact only with near neighbors, such as individuals within a certain Euclidian distance (metric models) or a set number of nearest neighbors regardless of distance (topological models) [38], [40]. These neighbors may change through time due to the motion of individuals (time-varying networks) [23], [35]–[36], [39]–[45]. For this class of opinion dynamics models, groups are typically highly cohesive, and the motion of groups is well approximated by simple majority rule when collectively deciding between discrete options (see supplemental text S1 and figures S1,S2). Effective consensus thus emerges from local interactions among individuals. Although individuals cannot explicitly ‘tally votes,’ they nevertheless exhibit the capacity to select, collectively, the direction preferred by the majority when conflicting preferences exist [23], [25], [43], even in the presence of a ‘strongly opinionated’ minority [23]. Consequently it is not necessary to simulate the full spatial dynamics to capture accurately the outcome of consensus decision-making by organisms [23].

One consequence of consensus decision-making (regardless of the precise mechanism by which consensus is achieved) is that it breaks the direct relationship between individual preference and action. An individual's preferences may be overridden by those of others, such that the individual experiences a part of the environment that it would not have had it been alone. This alters what individuals learn about their environment and also implies that learning by multiple grouping individuals becomes coupled; the preferences of one individual can affect what another experiences, and what one individual learns can affect the future learning of other individuals in the group. Social learning allows individuals of many social species to learn by observing the behaviors of conspecifics [46]–[52]. Individuals tend to follow the decisions of others when their personal information is unreliable [53] or costly to acquire [54]. Nonetheless, associative learning has not been investigated in a social context. Furthermore, the majority of experiments on social learning study a single test subject (the observer), separated from conspecific demonstrators (e.g. [53]–[59]). In a freely behaving group, however, each individual can simultaneously act as demonstrator and observer, resulting in a coupling between preferences, which potentially affects the learned behavior of all individuals in the group.

The impact of these coupled dynamics on associative learning in animal groups has yet to be explored, despite the fact that associative learning (whereby individuals learn to associate environmental cues with rewards), occurs in all organisms with a nervous system [60]–[61]. Since consensus decisions break the direct feedback between preference and experience, it is not clear to what degree learning is beneficial in a collective context, whether learning rules in a social context need to be more complex (such as group size or context dependent) in order to be effective, or how learning in isolation and subsequently pooling opinions as a group compares with learning as part of a collective.

**Figure 1:**
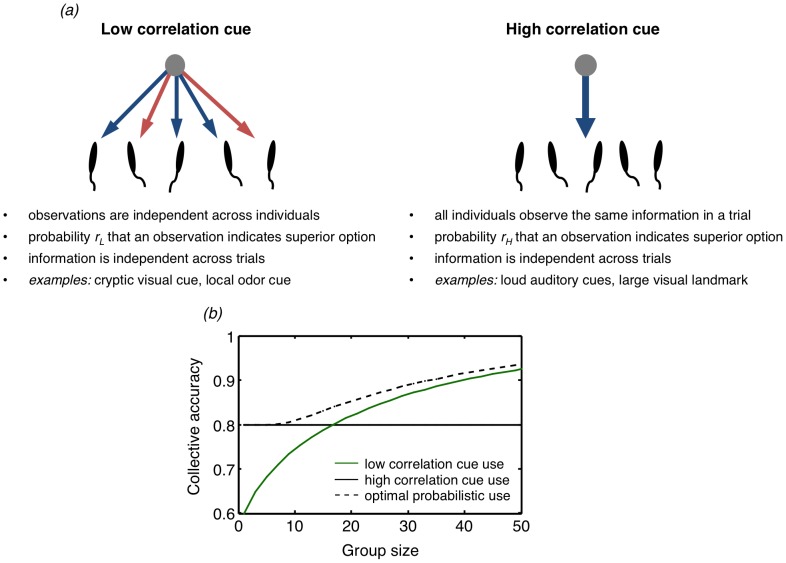
The observational correlation of cues. (a) Observational correlation describes the degree to which observations made by different group members are independent of each other. A low correlation cue provides group members with independent observations, while a high correlation cue provides just one observation to all group members on a given trial. (b) Exclusive use of a low correlation cue results in a monotonic increase in collective accuracy as group size increases (green solid line), a hallmark of collective wisdom (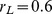). In contrast, exclusive use of a low correlation cue shows no increase in collective accuracy with group size (black solid line; 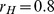). A mixed strategy, whereby individuals probabilistically choose one of the cues, may lead to collective accuracy greater than that obtained from using either of the cues exclusively when 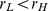.

Furthermore, natural environments typically contain not one, but potentially many informative cues, and a crucial challenge for animals is to learn the appropriate relative usefulness of the cues in order to maximize decision accuracy. Optimal voting theory [62] demonstrates that the relative value of environmental cues depends on group size as well as the properties of the cues. Similar to decision-making in isolation, the reliability of a cue (the probability that it accurately predicts a reward or punishment) is important. Unique to collective decision-making, however, and of central importance, is the observational correlation of a cue (the similarity between two individuals' observations) (figure 1) [63]. In nature, some cues may be subject to relatively low observational correlation, such as cryptic visual cues, where individuals exhibit a relatively independent probability of correctly observing accurate information from the cue [21]. Other cues, however, likely result in high correlation, such as loud auditory cues, strong environmental odors, or large visual landmarks that can readily be perceived by all individuals in the group. For high correlation cues, group members perceive similar observations of the cues, such that there is a high probability that they all receive true (or false) information (figure 1). Because correlations decrease the independence of observations made by different group members, they limit the benefits derived from aggregating observations [62]–[63]. In general, for group-living animals the optimal behavior is to rely primarily on those cues that are less correlated and those that more reliably lead to rewards [62].

**Figure 2:**
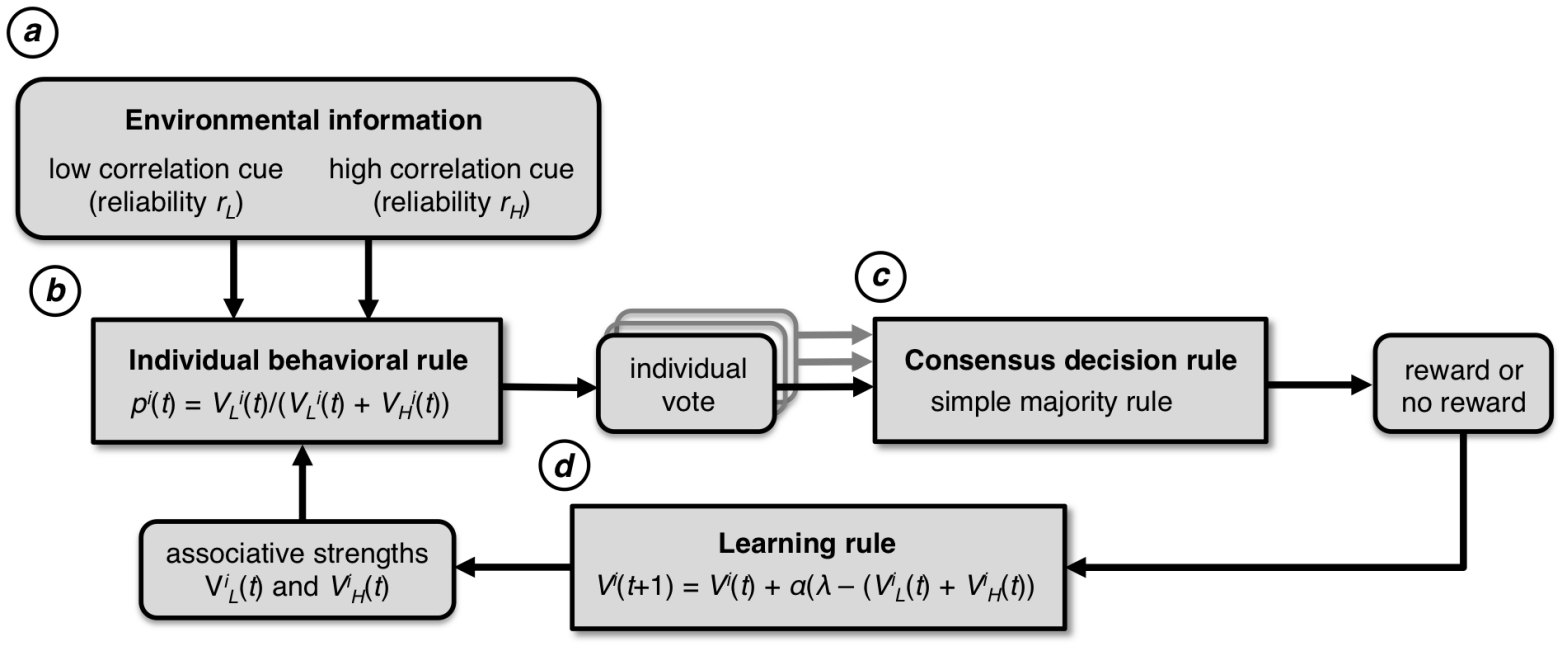
Flowchart of the collective learning process. (a) A decision trial begins with individuals observing cues in the environment. In this model we have two cues, one with low observational correlation and one with high correlation. (b) Individuals use the low correlation cue with probability 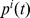 and the high correlation cue with probability 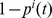 in order to form a discrete vote for one of the two options. In the case that both cues indicate the same option, the individual always votes for that option. (c) The votes are aggregated and a consensus decision is made by simple majority rule. (d) The resulting reward or punishment is used to update each individual's voting behavior. A learning rule similar to the Rescorla-Wagner learning rule is used to update the associative strength(s) of the cue(s) that were present at the chosen option. The associative strengths determine 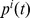 and therefore how an individual votes.

Here we present a general framework for studying collective learning and consensus decision-making in animal groups (figure 2). In this framework, we simulate individual associative learning as in the existing literature, i.e., we do not make any new assumptions regarding the mechanisms by which individuals learn to associate cues with rewards, nor do we afford additional cognitive abilities to individuals. However, we place individual learning within the context of consensus decision-making, as exhibited by many self-organized animal groups [15], [32]–[34]. Our framework is agnostic to the mechanism by which animal groups reach consensus, and thus our conclusions are consistent with both spatial and non-spatial models of collective decision-making. This allows us to focus on the coupled dynamics between consensus decisions and associative learning, a previously unexplored aspect of animal collective behavior.

## Model

We consider a group of 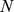 individuals choosing between a number of discrete options. Here we model two options (which we denote as option A and option B), for simplicity, and because in nature many decisions are binary (such as whether or not to flee from a potential predator, whether or not to approach a shelter, or selecting among potential areas in which to forage). In a given trial, either option is equally likely to be superior *a priori* (a uniform prior over the options); therefore, all of the information available to individuals is contained in the environmental cues. We assume that the individuals in the group employ consensus decision-making, and, for tractability, that the reward (e.g., safety from a predator) is shared equally among individuals in the group.

### (a) The informational environment

In order to gain information about the quality of the two options, individuals observe environmental cues, which could be odor, visual, auditory, or other sensory cues (figure 2a). Each cue indicates to each individual that one or the other option is superior on that particular trial. For simplicity we assume two such cues. Since individuals in groups must differentiate between cues with different degrees of observational correlation in order to improve collective accuracy [62], in our model one of the cues has low correlation (i.e., observations of the cue by individuals in the group are independent of each other, such that individuals may have opposing information from this cue regarding which is the superior option), while the other cue has high correlation (i.e., all individuals make the same observation of the cue and agree about which option this cue indicates is superior; figure 1) [63]. The reliability of a cue is the probability that it correctly predicts the superior option. These reliabilities are denoted by 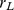 and 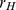 for the low and high correlation cues, respectively, and can range from 0.5 to 1. Effective collective learning would allow individuals in groups to give additional value to the low correlation cue (beyond its reliability), due to the benefit of multiple independent observations that cue affords. Consequently, the most interesting scenario is that in which one cue has lower correlation while the other cue has higher reliability, i.e., 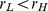.

### (b) Individual decision-making

Individuals translate their observations of the two environmental cues into a discrete preference, or vote, for one of the two options. Following well-established psychological models of decision-making by isolated individuals, we assume that the individual rule is to vote for an option with a probability proportional to the sum of the associative strengths (see below) of all of the environmental cues that indicate that option. Thus, individuals vote for option A with a probability 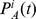 and option B with probability 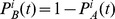, where 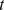 is the current trial, and 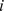 indicates a particular individual. In our model, because we have only two cues and two options, from the perspective of an individual there are only two possible scenarios: either the two cues both indicate the same option is superior, or they indicate different options. When the two cues indicate that the same option is superior, the voting rule implies that an individual always votes for that option. However, when the two cues indicate opposing options, an individual votes for the option indicated by the low or high correlation cue with a probability proportional to the associative strength of that cue. We denote the probability of choosing the option indicated by the low correlation cue as 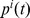, irrespective of whether the low correlation cue indicated option A or option B. Similarly, the probability that an individual votes for the option indicated by the high correlation cue is 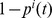 (figure 2b).

### (c) Consensus decision-making

Once individuals have formed an opinion about which option they consider superior, these opinions must be aggregated in order to produce a collective decision. For some species, social interactions are weak and temporary. Other species, however, are strongly social, and empirical work has shown that, despite employing different interaction rules, many animal species, including primates [28]–[29], meerkats [30], fish [16]–[18], [20], [23], and insects [20], [27], typically make consensus decisions. Our model does not consider the precise mechanism by which individuals interact, since here it is the outcome of consensus decision-making, and its relationship to individual associative learning, that is important (however, we demonstrate, in supplemental text S1 and figure S2, that considering the specifics of interactions, such as by simulating local spatial interactions among individuals [23], [41], [43], does not affect our conclusions). Based on experimental evidence from many types of animal groups [17]–[18], [20]–[21], [23], [32]–[33] we assume that individuals can, and often do, select the option preferred by the majority (figure 2c). Further empirical and theoretical work has demonstrated that the presence in the group of individuals with no preferences can even strengthen majority rule in animal groups [23]. As shown by spatially-explicit models of mobile animal groups and in experiments, when there are equal numbers of votes for each option, the group is able to avoid a deadlock and chooses an option randomly [23], [25], [43] (supplemental text S1 and figure S2).

### (d) Individual learning

After the group chooses one of the options, individuals experience the outcome (the presence or absence of a reward) and employ an associative learning rule to update their knowledge of the environment based on this experience (figure 2d). Following standard models of learning in the psychology literature, knowledge of the environment is encoded by an ‘associative strength’ for each environmental cue. Each individual 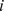 stores two associative strengths, 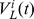 and 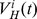, representing, respectively, the individual's valuation of the low and high correlation cue.

Individuals in our model do not explicitly estimate the size of the group they are in, nor the observational correlation or reliabilities of the cues, which all contribute to determining the optimal voting behavior (see below). It is not known to what extent animals are aware of the size of the group to which they belong, and it is likely that many animals under consideration in this model are unable to accurately estimate group size, either because of limited cognitive abilities, because the group may be large or fluctuating, making estimates of its size difficult, or because of the local nature of interactions [64]. Similarly, it is not known whether individuals can estimate the observational correlation of cues; therefore, in this model we employ a conservative approach, and assume that they are unable to do so (also, as we will show, they need not be able to do so). In short, we do not make new assumptions about the process by which associative learning occurs [3]–[6].

At the start of a simulation, all individuals lack any knowledge of the two cues and therefore the associative strengths for both cues are identical and very small 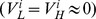. Also, following standard models of learning, individuals update the associative strength(s) only of the cue(s) that indicated the option that was ultimately selected by the group. Associative strengths of cues are updated according to the following learning rule, which is similar to the well-known and experimentally-validated [3]–[6], [65]–[69] Rescorla-Wagner rule: 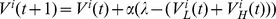, where 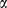 is the learning rate (here taken to be 0.1), 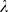 is 1 if the option selected by the group was the superior option and 0 if it was not [5], [7], and 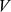 represents the associative strength of any cue that indicated the option chosen by the group. In general, this individual learning rule increases the associative strength of cues that are consistently paired with a positive outcome (the superior option) and decreases those that are paired with a negative outcome (the inferior option) and therefore serves as a memory of past events. Because individuals observe independent and potentially different information from the low correlation cue, only a fraction of the group will update the associative strength for that cue on a given trial. This results in individuals in a group potentially learning different associative strengths for the cues despite sharing a common experience of decision outcomes. The associative strengths are related to the voting behavior in the following way: 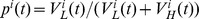. Equivalently, individuals vote for an option proportionally to the total associative strength of the cues they perceive as indicating that option. This linear mapping between associative strengths and voting behavior is common in models of learning [7], although we explore alternate mappings and demonstrate in the supplemental text S2 and figure S3 that this does not impact the results.

During the course of repeated trials, an individual's associative strengths are modified, leading to a change in its probabilities 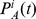 and 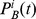 of voting for the two options, which are the direct determinants of the group's resulting decision accuracy. We simulate learning dynamics for a wide range of group sizes 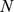 and across all combinations of cue reliabilities 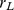 and 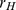 in order to assess how collective learning functions under different conditions.

### (e) Model extensions

In the model framework presented (figure 2), we have deliberately made biologically realistic but relatively simple assumptions. However our model is robust to deviations from these assumptions. For example, as we show in supplemental text S3 and figure S4, the general conclusions we arrive at do not depend on the exact choice of the collective decision rule by which consensus decision-making is achieved, nor on the specific individual voting rule (linear or nonlinear) (supplemental text S2 and figure S3). In addition, though not addressed here, the model framework can readily be tailored to generate predictions about specific behavioral contexts or animal species, including species in which consensus is not strongly enforced, or in which individuals have varying degrees of influence in the group decision, due to behavioral syndromes, differing physiological needs, or dominance hierarchies. Alternate learning rules may also be studied. The core ingredients are merely that (1) individual experiences are influenced by other group members and (2) learning occurs with regard to the experienced outcome, not the individually preferred one.

## Results

### (a) Collective learning across environments

**Figure 3:**
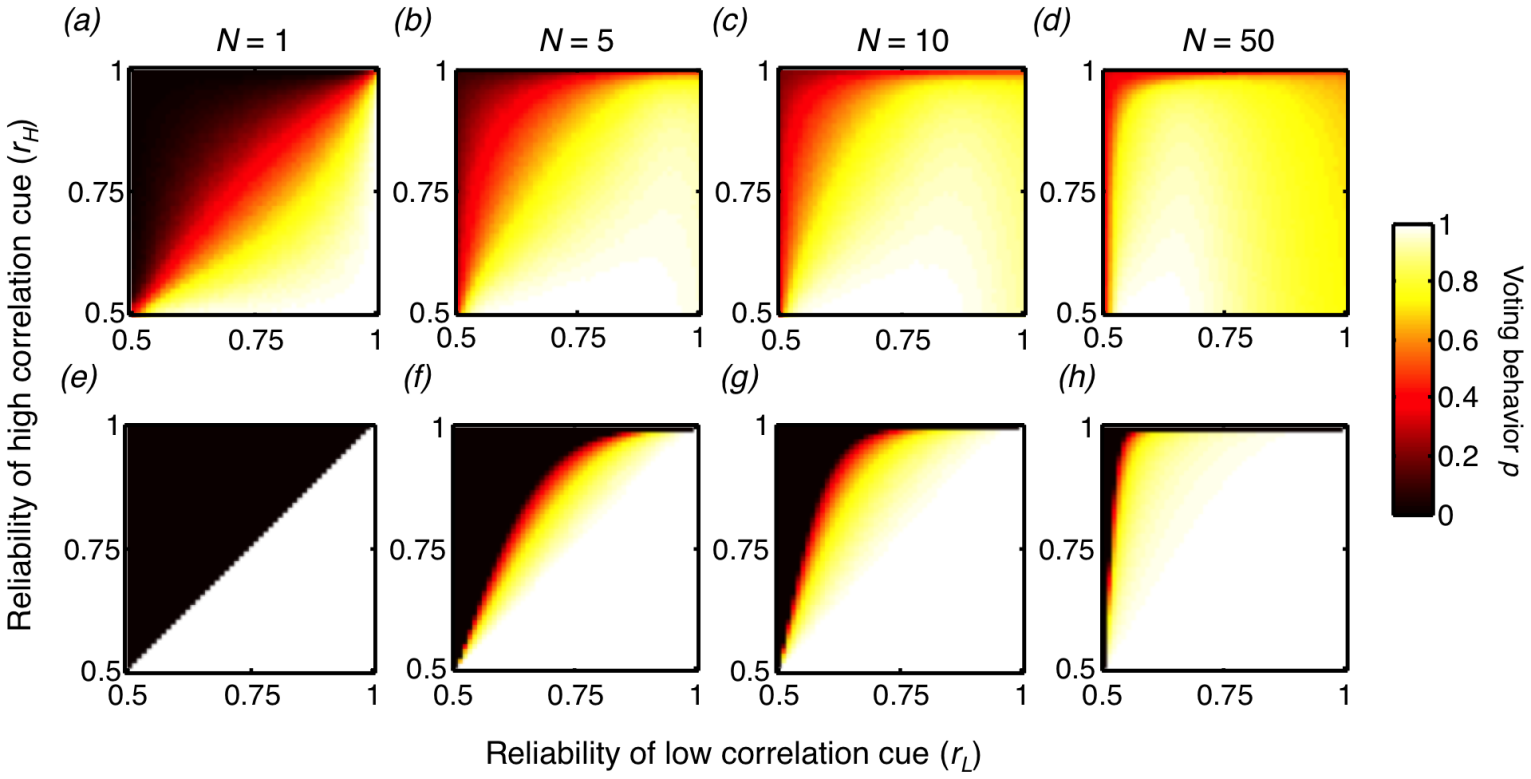
The learned and optimal behavioral strategies of individuals in a social context, across environmental conditions and group sizes. (a–d) The mean learned voting behavior 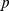 (the probability that individuals use the low correlation cue), for all combinations of reliabilities of the low correlation cue (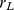) and high correlation cue (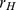) for (a) group size 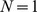 (isolated case), (b) 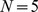, (c) 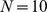, and (d) 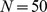. For each environment and group size combination, 500 simulations of 1000 training trials were performed, using a learning rate of 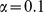, and the mean behavior of the last 100 trials across the simulations is reported. (e–h) The optimal voting behavior for the environments and group sizes shown in (a–d).

In the case of non-social animals, or those in isolation 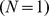, if both cues indicate that the same option is superior, maximizing reward rate requires an individual to choose that option. However, if the two cues indicate that different options are superior, then the individual should choose the option indicated by the more reliable cue: 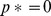 if 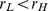 and 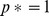 if 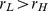 (where asterisks denote the optimal behavior). If we simulate such a case, we find that isolated individuals do learn to give greater weight to the option indicated by the more reliable cue, such that 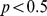 when 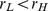 and 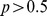 when 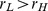 (figure 3a). This result is compatible with previous experiments on isolated animals [70]–[71].

If the collective learning process is unaffected by the observational correlation of cues or group size, we might expect the learned voting behavior of individuals in groups 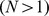 to be identical to that of isolated individuals. This is not what we observe. As group size increases, the learned voting behavior changes, such that individuals rely more heavily on the low correlation cue for a given environment (figure 3b–d), indicative of effective collective learning. For relatively large groups 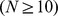, individuals rely primarily on the high correlation cue only when that cue is extremely reliable (figure 3c–d).

Consensus decision-making therefore results in learned individual voting behavior that is markedly different from that exhibited by isolated individuals under identical environmental conditions. We find that the coupling between the learning of group members allows individuals to incorporate observational correlations, reliabilities, and group size into their valuation (associative strength) of the cues in a way that allows them to make substantially more accurate consensus decisions.

### (b) The optimality of collectively learned behavior

The above results demonstrate that grouping individuals exhibit learned voting behavior that depends not only on cue reliability, but also on the observational correlation of environmental cues, as well as group size (without requiring them to be able to estimate any of these explicitly). However, it is not clear, given the environmental conditions, how close the resulting performance is to that which is optimal. To investigate this, we derived the optimal individual voting behavior that maximizes collective accuracy, for any environmental condition (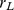 and 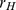) and group size 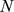. In the case where both cues indicate that the same option is superior, an individual should vote for the indicated option. However, when the two cues indicate that different options are superior, the optimal behavior is to vote for the option indicated by the low correlation cue with probability 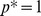 when its reliability is greater than that of the high correlation cue 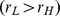, 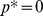 when the high correlation cue is very reliable 
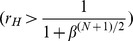
, and otherwise 
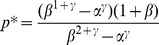
, where 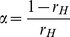, 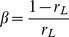, and 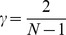 (see supplemental text S4 for the complete proof). In short, when the two cues indicate different options are superior, the optimal voting behavior is to choose exclusively the option indicated by the high or low correlation cue if its reliability is sufficiently high, and otherwise to exhibit a mixed strategy in which individuals probabilistically choose either option (figure 3e–h).

**Figure 4:**
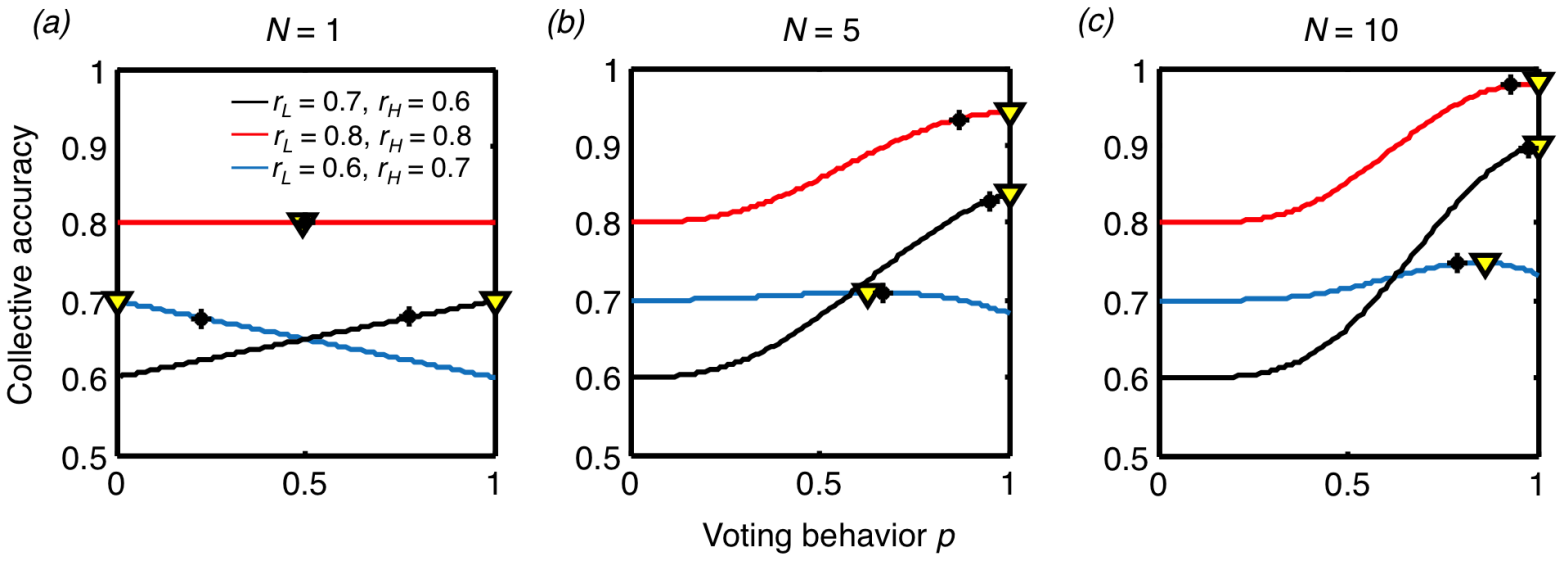
The accuracy landscape of collective decisions. Lines denote the collective accuracy as a function of voting behavior *p* for three representative environments and for group size (a) 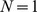, (b) 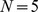, and (c) 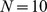; yellow triangles denote the optimal voting behavior that results in the maximum collective accuracy; black stars represent the voting behavior learned by our model for that group size and environment. As group size increases, it is optimal to rely increasingly on the low correlation cue, regardless of the environmental contingencies. The learned behavior is able to track this shift in the optimal behavior, resulting in near-optimal accuracies for any group size and environment.

We illustrate how the collective accuracy varies with the individual voting behavior for a range of environmental conditions and group sizes, and we show the optimal voting behavior (yellow triangles) and the learned voting behavior (black stars) on this landscape (figure 4). When 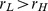 (black lines), it is always optimal to choose exclusively the option indicated by the low correlation cue regardless of group size. When 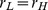 (red lines), individuals in isolation 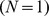 should value the two cues equally but, in groups, should rely exclusively on the low correlation cue. When 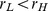 (blue lines), individuals in isolation 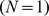 should choose exclusively the option indicated by the high correlation cue. However, as group size increases, the optimal behavior gradually shifts towards greater reliance on the low correlation cue. In all cases, we observe that the learned behavior closely tracks the optimal behavior (figure 4).

**Figure 5:**
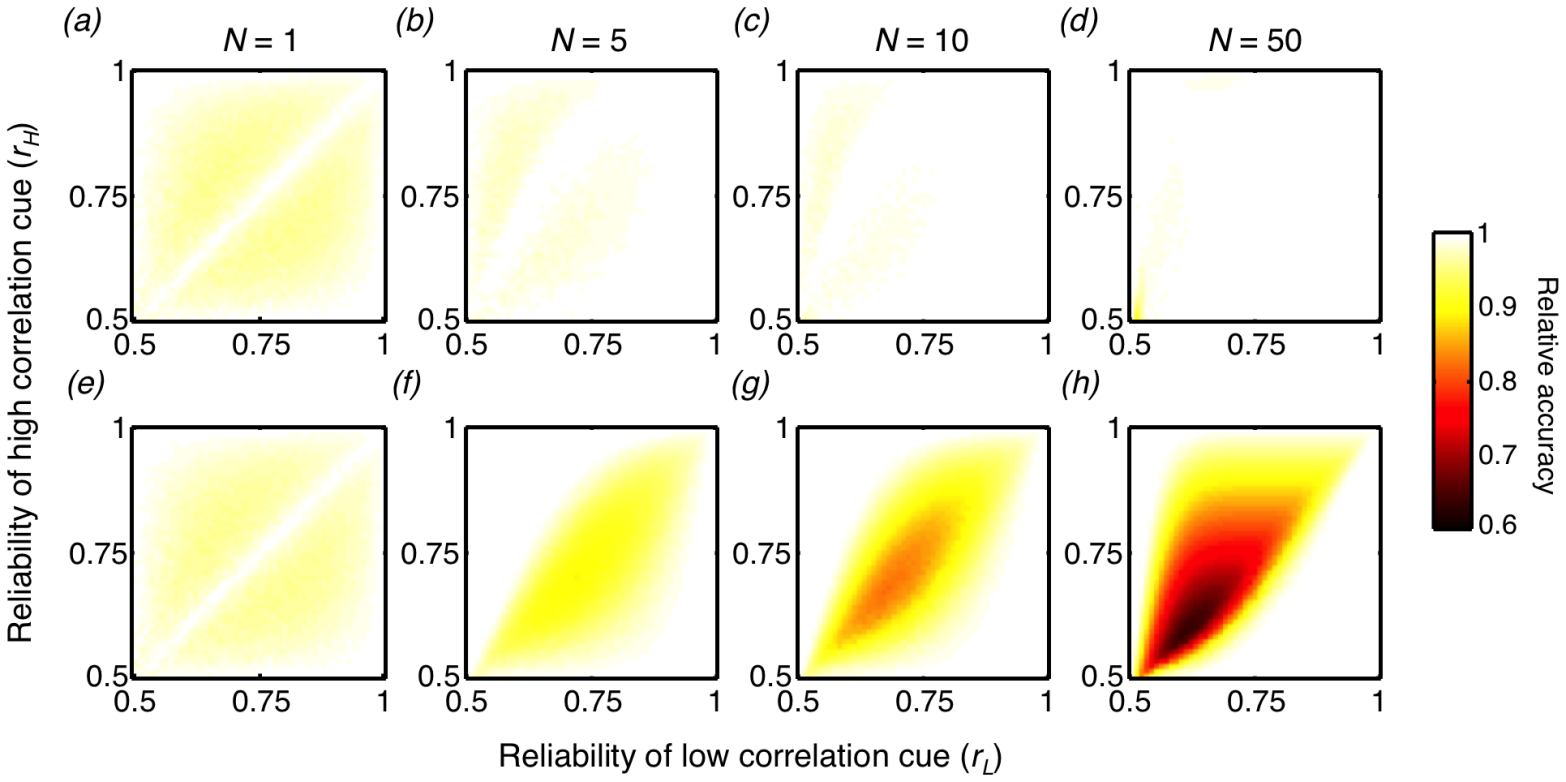
Comparing the accuracy of collectively and individually learned behavior to the accuracy of the optimal behavior. (a–d) The collective accuracy resulting from the collectively learned behavior as a fraction of the maximum possible for that environment and group size, for all combinations of reliabilities of the two cues, for group size (a) 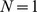, (b) 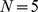, (c) 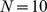, and (d) 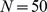. For each environment and group size combination, 500 simulations of 1000 training trials were performed, using a learning rate of 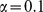, and the mean behavior of the last 100 trials across the simulations is reported. (e–h) The collective accuracy resulting from the behavior learned in isolation as a fraction of the maximum possible for that environment and group size.

We generalize this result by showing the collective accuracy as a result of the collectively learned voting behavior for all environments and a wide range of group sizes (supplemental figure S5). We further show this accuracy as a fraction of the maximum possible accuracy, achieved by the optimal voting behavior (figure 5a–d) and find that across all conditions, the achieved accuracy is extremely close to the maximum possible.

An implicit assumption in studies of collective intelligence is that the ‘wisdom of crowds’ accrues due to individuals pooling knowledge that was learned independently. If true, then one would expect individuals that exhibit a voting behavior learned in isolation, and whose opinions are subsequently pooled into a group decision, to also exhibit a high degree of collective intelligence. In fact, we find that such groups perform relatively poorly (figure 5e–h), and do so increasingly for larger group size. Therefore, we find that it is not sufficient for individuals to learn in isolation and to subsequently pool their knowledge. Instead, it is important for individuals to incorporate observational correlation and group size into their valuation of a cue, for which collective learning is critical.

In this model, and based on theoretical and empirical evidence [17]–[18], [28], [34], we assumed that animal groups make decisions through simple majority rule. However, this is only one particular method of aggregating opinions. For example, an alternative consensus decision rule is to decide on an option only if a minimum proportion of the group votes for that option (<50% or >50%, representing submajority and supermajority thresholds, respectively). This may occur in the context of predator detection where, because of asymmetric costs, the reaction of a small proportion of the group may cause the entire group to flee [30], [72]. Limiting a group to simple majority rule, rather than the more general sub- and supermajority thresholds, could, in principle, constrain the accuracy that a group may attain. The provably optimal voting rule for groups in which members indicate their vote, explicitly count votes, and can adopt any type of vote aggregation rule (capabilities unlikely to be available to most animal groups) was found by Nitzan and Paroush [62], in the context of human organizations such as juries, governing bodies, and medical panels. They found that the optimal strategy is to adjust the majority threshold according to the cue reliabilities and group size.

We compared the efficacy of this ‘globally optimal’ group consensus decision rule to the optimal individual voting behavior with the constraint of simple majority rule that we identified and found that the two rules result in nearly identical collective accuracies (supplemental text S5 and figure S6). Therefore, the decentralized rule that many animals follow, in which a probabilistic individual behavior is employed instead of a global supermajority rule, poses very little restriction on the collective accuracy that can be achieved by groups.

### (c) Collective learning in dynamic environments

That the collectively learned individual voting behavior substantially outperforms the behavior learned in isolation and subsequently expressed in a group context suggests that collective learning may play an important role in group-living. However, many animals exist in ‘fission-fusion’ populations in which groups readily merge and split over a period of weeks [73], days [74] or even minutes [75]–[81]. Thus individuals may not repeatedly learn about their environment with the same group members or in the same group size. Furthermore, natural environments are dynamic, and cues can change in their reliability in predicting the location of food or predators (for example, food availability may be seasonal). In order for individuals to make accurate decisions in this setting, collective learning must be robust to the splitting and merging of groups, and to changes in cue reliability.

We first suppose that individuals employ the optimal individual voting behavior for their environment and group size and consider abrupt changes in group size and environmental conditions. The resulting accuracy experienced in the new environment is compared to the accuracy that would result from using the appropriate optimal voting behavior for the new environment. Across changes in group size (supplemental figure S7a–c) and environmental conditions (supplemental figure S8a–c), we find that subsequent to most changes in group size and environmental conditions, the collective accuracy remains close to optimal, even when learning has not occurred in the new context. This is because it is optimal to rely on the low correlation cue for most environments and group sizes (figure 3e–h), so that changes within that regime do not result in substantial decreases in accuracy. We find that individuals are far from optimal only when there are large changes in group size (particularly when many small groups are combined into a very large group, or vice-versa) or when the reliability of environmental cues changes drastically.

We selected several particularly challenging environmental transitions and subjected our collective learning model to these conditions. Individuals across all contexts are able to adaptively adjust their voting behavior subsequent to a change in group size or environment and reach an accuracy that is close to the maximum possible for the new context (supplemental figure S7d–f, S8d–f). Learning in one environment does not preclude learning in a new environment, nor does the collective context impede adapting to changing environments. Thus, fission-fusion dynamics do not necessarily limit the ability of animals to locate the effective voting behavior across a wide range of group sizes.

## Discussion

To date, studies of associative learning have largely been informed by experiments on individuals in isolation. Under such circumstances, there is direct feedback between preference and experience that often allows individuals to accurately learn the value of cues in the environment. However, many organisms spend at least part of their lives in groups and in order to maintain the benefits of group living, often must make consensus decisions. Coming to a consensus decouples the direct relationship between individual preferences and the outcomes of decisions, and it is not clear how animals could learn an accurate valuation of environmental cues.

Here we demonstrate that embedding simple associative learning in a social context fundamentally alters what individuals learn about their environment and spontaneously allows organisms to achieve close to provably optimal collective decision-making, regardless of environmental conditions. This is in contrast to individuals who learn in isolation and subsequently pool information as a group, which can result in relatively poor collective decision-making when cues have varying degrees of observational correlation.

We show that the individual behavior that maximizes collective decision accuracy is a function of both group size and the properties of environmental cues (notably their reliability and the observational correlation between individuals). However, when learning collectively, individuals are able to accurately value environmental cues without explicitly estimating any of these parameters. Thus, sophisticated cognitive processes are not necessary for highly effective decision-making in a wide range of environments.

While our results are robust to relaxing several of the model assumptions (see supplemental text S2,S3 and figures S3,S4,S7,S8), our model framework can also be applied to other classes of collective decision-making mechanisms [82]–[90]. For example, it is plausible that learned knowledge of the environment (encoded by the associative strengths) may translate into influence in the group decision, whereby individuals with stronger opinions about which option is superior may have greater influence [91]–[92]. Furthermore, many groups are composed of dominance hierarchies with a small subset of individuals controlling the group decision [24], individuals in groups may have intrinsically different leadership abilities due to behavioral syndromes [93], and individuals may have different physiological needs [94]. These may all contribute to differential influence in the group decision and consequently alter what is learned by group members. These modifications, which may more accurately model particular animal species, are interesting avenues of future research given their potential effect on collective learning in animal groups.

In our model we assumed that an individual's learning rule is similar to that found in animals learning in isolation. This assumption precluded an individual from directly detecting the observation correlation of cues or the size of the group, parameters that we showed to be important in the determination of the optimal behavior. Nonetheless, even if individuals were not afforded additional cognitive or communication abilities, they were able to learn near optimal behavior. However, it is possible that the learning rule is indeed different for animals in a collective context. Our work suggests the need for empirical work that studies how associative learning functions within animal groups.

We have considered a simple, and potentially ubiquitous, form of collective learning, in which individuals' experiences of the environment is biased by the experiences of others. The same learning rules that are known to lead to effective decision-making in single individuals are shown to be equally effective in groups of any size. This affords social organisms a robust and simple mechanism for learning behaviors that lead to accurate decisions in relatively complex environments containing multiple cues that vary in reliability and observational correlation, and which may fluctuate in time. Therefore, collective learning may allow even simple group-living organisms to reliably achieve collective wisdom across diverse environmental and social contexts.

## Supporting Information

Figure S1
**Illustration of the zones of interaction in the spatial model.** Individuals are repelled by any neighbors found in the inner zone (with radius 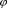) and this repulsion force takes precedence over any other social forces or innate preferences. Individuals are attracted to, and align with, neighbors within the outer zone (with radius 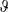). Individuals cannot detect others outside of this outer zone.(TIFF)

Figure S2 **Comparing the behavior of the spatial schooling model to the assumptions of simple majority rule.** *(a–c)* The proportion of trials in which a given fraction of the group reached target A, when half of the group prefers target A and the other half prefers target B. In simulated groups, either none or all of the individuals reach target A, demonstrating a high degree of group cohesion. Shown is the result of 10000 simulated decision-making bouts for each group size. *(d–f)* The proportion of trials that the group arrives at target A when a given fraction of the group prefers target A. The group tends to arrive at target A only when more than half of the group prefers target A, which agrees with simple majority rule. Shown is the result of 1000 simulated decision-making bouts for each fraction of the group and group size.
(TIFF)

Figure S3
**Collective learning for a range of logistic voting behavioral rules.** Top row illustrates different steepnesses of the logistic function used for the voting behavior, from very shallow (left) to very steep (right). Bottom row shows the resulting collective accuracy (as a fraction of the maximum possible accuracy for that environmental condition and group size) as a function of the steepness of the voting rule. All possible combinations of group sizes 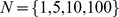, 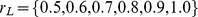, and 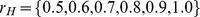 were tested. For each combination, 1000 simulations were performed for 1000 training trials using a learning rate of 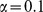, and the mean collective accuracy of the last 100 trials across all simulations was calculated. Collective learning suffers at very shallow logistic functions for the number of trials but performs equivalently well at sufficiently steep functions.(TIFF)

Figure S4
**The learned and optimal voting behavior of individuals in a collective context, across environmental conditions and group sizes, for groups employing a logistic consensus decision rule.** *(a–d)* The mean learned voting behavior, or probability 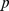 that individuals vote for the option indicated by the low correlation cue, for all combinations of reliabilities of the low correlation cue (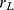) and high correlation cue (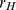) for *(a)* group size 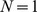 (isolated individuals), *(b)* 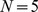, *(c)* 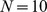, and *(d)* 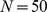. For each environment and group size combination, 500 simulations of 1000 training trials were performed, using a learning rate of 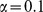, and the mean behavior of the last 100 trials across the simulations was plotted. *(e–h)* The optimal voting behavior for the environments and group sizes shown in *(a–d)*.(TIFF)

Figure S5
**The collective accuracy resulting from the collectively learned behavior.** *(a–d)* The mean collective accuracy for all combinations of reliabilities of the two cues, for *(a)* group size 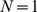, *(b)* 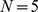, *(c)* 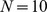, and *(d)* 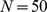. For each environment and group size combination, 500 simulations of 1000 training trials were performed, using a learning rate of 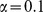, and the mean behavior of the last 100 trials across the simulations was used.(TIFF)

Figure S6
**Comparison of collective accuracy resulting from the optimal voting rule with the constraint of simple majority rule to the accuracy attained when any group decision rule can be employed (the ‘global’ optimal rule).** For each group size, we tested all combinations of cue reliabilities 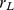 and 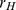 and calculated the fraction of the accuracy of the globally optimal rule that the simple majority optimal rule achieves. Across all group sizes and environments, the simple majority optimal rule nearly always achieves greater than 99% of the accuracy of the globally optimal rule.(TIFF)

Figure S7 **Collective learning subsequent to abrupt changes in group size for three representative environments.** *(a–c)* We assume that individuals use the voting behavior that is optimal for the environment and starting group size (y-axis) and calculate the difference in collective accuracy that results from using that behavior in a range of new group sizes (x-axis) relative to the optimal behavior for the new group size. *(d–f)* We select four of the most challenging conditions in each environment (colored dots in *a–c*) and simulate collective learning in those contexts. Colors of lines match the dots in *(a–c)*. Following the change in group size (which occurs after 500 trials), individuals in all conditions asymptote at close to the maximum possible for the new context.
(TIFF)

Figure S8
**Collective learning subsequent to abrupt changes in the reliability of environmental cues for three representative group sizes.** *(a–c)* For simplicity, we fix the reliability of the high correlation cue at 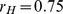 and consider all combinations of changes in the reliability of the low correlation cue. We assume that individuals use the voting behavior that is optimal for the starting environment and group size (y-axis) and calculate the difference in collective accuracy that results from using that behavior in a range of ending reliabilities of the low correlation cue (x-axis) compared to the optimal behavior for that environment. *(d–f)* We select four of the most challenging conditions in each group size (dots in *a–c*) and simulate collective learning in those contexts. Colors of lines match the dots in *(a–c)*. Following the change in cue reliability (which occurs after 500 trials), individuals in all conditions asymptote to close to the maximum possible for the new context.(TIFF)

Text S1 **Comparing simple majority rule to a full spatial model of collective decision-making.** Description of the spatial schooling model and comparison of its behavior to our assumptions of consensus and simple majority rule.
(PDF)

Text S2 **Relaxing the assumption of linear voting behavior.** Comparison of the collective accuracy resulting from a family of logistic (non-linear) voting behavior rules.
(PDF)

Text S3 **Relaxing the assumption of simple majority rule.** Comparison of the learned behavior resulting from a family of logistic (non-linear) collective decision rules.
(PDF)

Text S4 **Proof of the optimal voting behavior for animal groups.** Detailed derivation of the optimal voting behavior.
(PDF)

Text S5 **Comparing the optimal restricted voting rule to the globally optimal voting rule.** Comparison of the individual-level optimal voting rule to the globally optimal voting rule reveals little loss of collective accuracy.
(PDF)
