# Supplementary figures and images for: Collective Learning and Optimal Consensus Decisions in Social Animal Groups
Source: PLoS Comput Biol. 2014 Aug 7;10(8):e1003762. doi: 10.1371/journal.pcbi.1003762 (PMC4125046; doi:10.1371/journal.pcbi.1003762)

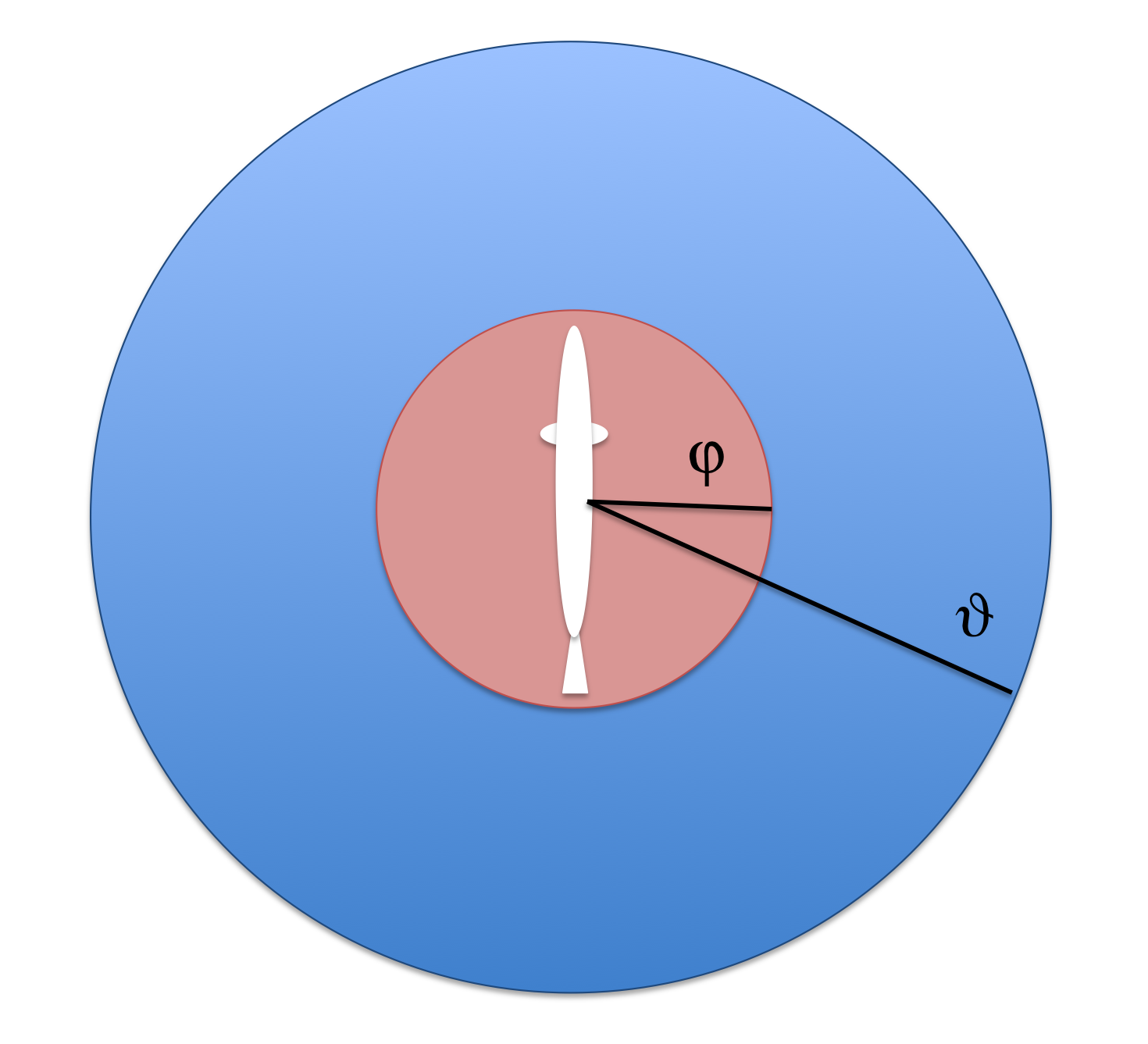

Supplement: Figure S1 — Illustration of the zones of interaction in the spatial model. Individuals are repelled by any neighbors found in the inner zone (with radius ) and this repulsion force takes precedence over any other social forces or innate preferences. Individuals are attracted to, and align with, neighbors within the outer zone (with radius ). Individuals cannot detect others outside of this outer zone. (TIFF) [file pcbi.1003762.s001.tiff]

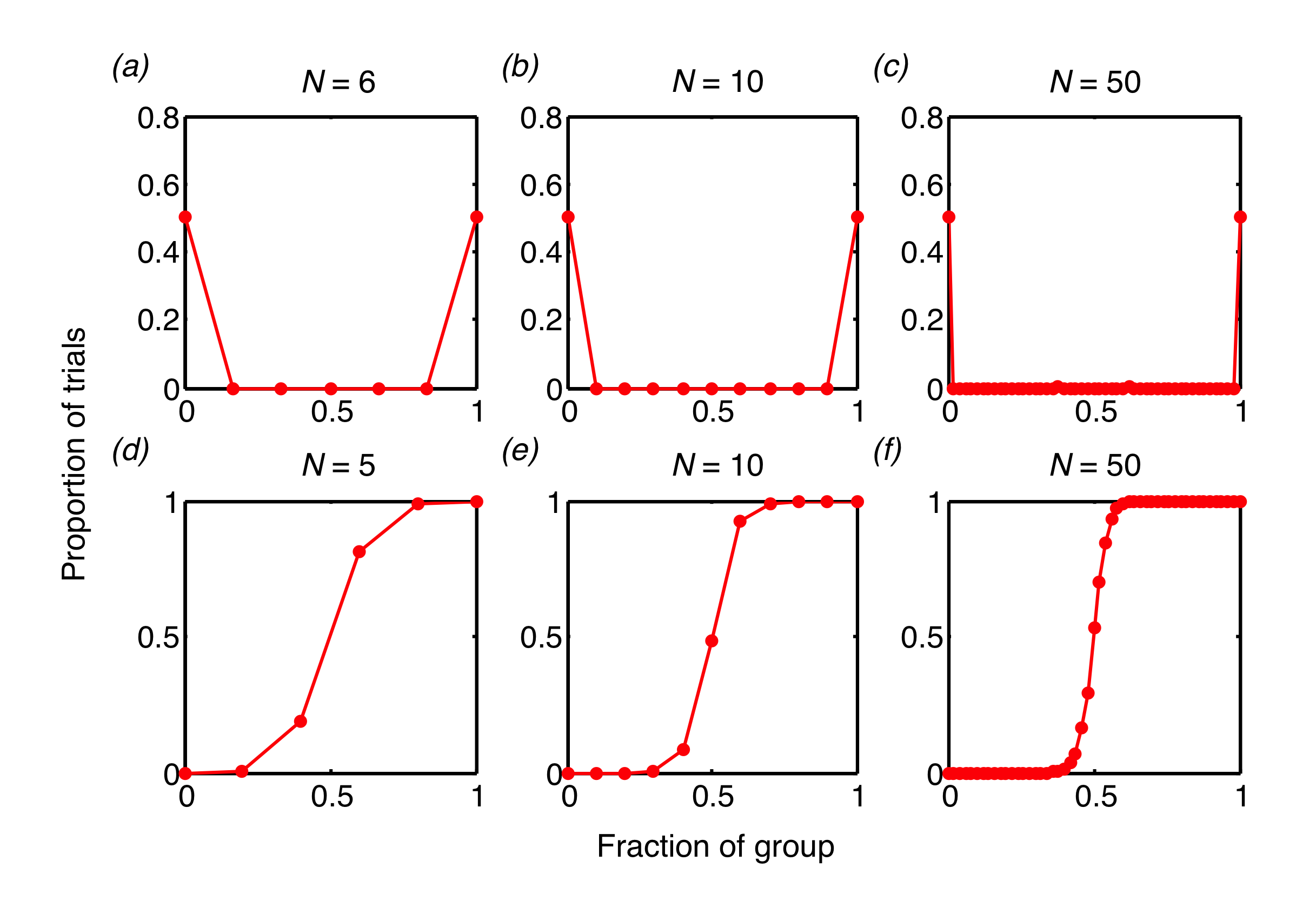

Supplement: Figure S2 — Comparing the behavior of the spatial schooling model to the assumptions of simple majority rule. (a–c) The proportion of trials in which a given fraction of the group reached target A, when half of the group prefers target A and the other half prefers target B. In simulated groups, either none or all of the individuals reach target A, demonstrating a high degree of group cohesion. Shown is the result of 10000 simulated decision-making bouts for each group size. (d–f) The proportion of trials that the group arrives at target A when a given fraction of the group prefers target A. The group tends to arrive at target A only when more than half of the group prefers target A, which agrees with simple majority rule. Shown is the result of 1000 simulated decision-making bouts for each fraction of the group and group size. (TIFF) [file pcbi.1003762.s002.tiff]

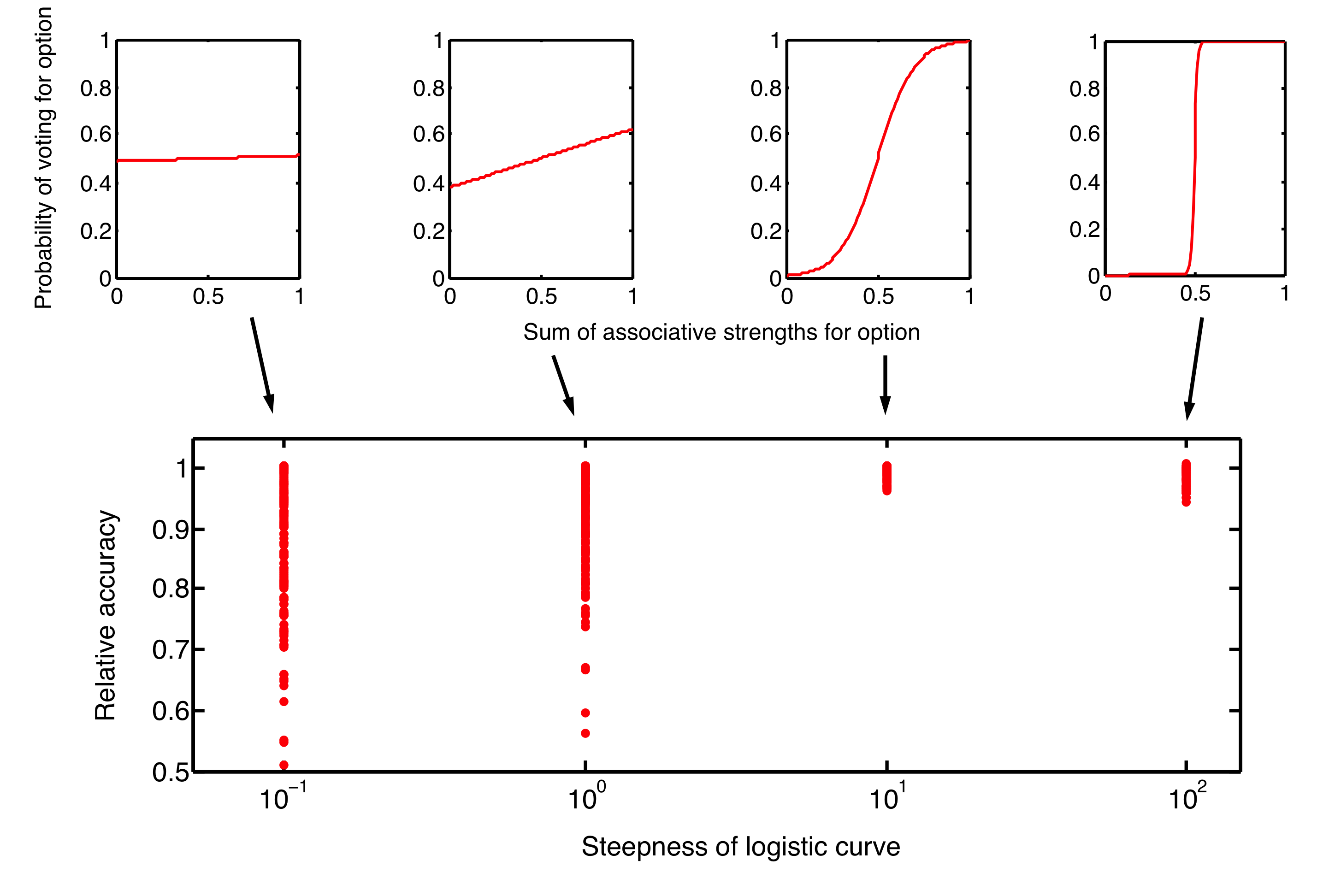

Supplement: Figure S3 — Collective learning for a range of logistic voting behavioral rules. Top row illustrates different steepnesses of the logistic function used for the voting behavior, from very shallow (left) to very steep (right). Bottom row shows the resulting collective accuracy (as a fraction of the maximum possible accuracy for that environmental condition and group size) as a function of the steepness of the voting rule. All possible combinations of group sizes , , and were tested. For each combination, 1000 simulations were performed for 1000 training trials using a learning rate of , and the mean collective accuracy of the last 100 trials across all simulations was calculated. Collective learning suffers at very shallow logistic functions for the number of trials but performs equivalently well at sufficiently steep functions. (TIFF) [file pcbi.1003762.s003.tiff]

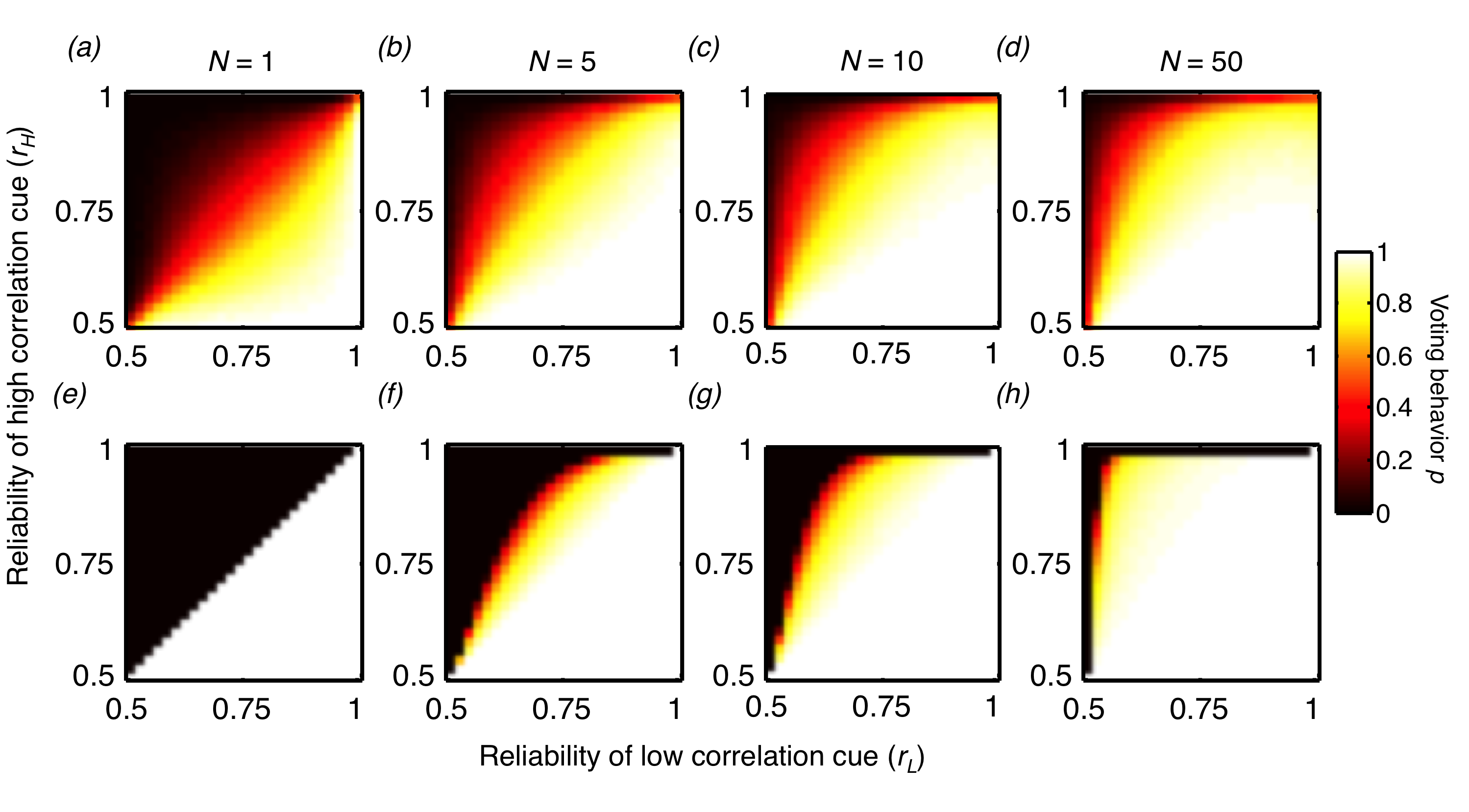

Supplement: Figure S4 — The learned and optimal voting behavior of individuals in a collective context, across environmental conditions and group sizes, for groups employing a logistic consensus decision rule. (a–d) The mean learned voting behavior, or probability that individuals vote for the option indicated by the low correlation cue, for all combinations of reliabilities of the low correlation cue () and high correlation cue () for (a) group size (isolated individuals), (b) , (c) , and (d) . For each environment and group size combination, 500 simulations of 1000 training trials were performed, using a learning rate of , and the mean behavior of the last 100 trials across the simulations was plotted. (e–h) The optimal voting behavior for the environments and group sizes shown in (a–d). (TIFF) [file pcbi.1003762.s004.tiff]

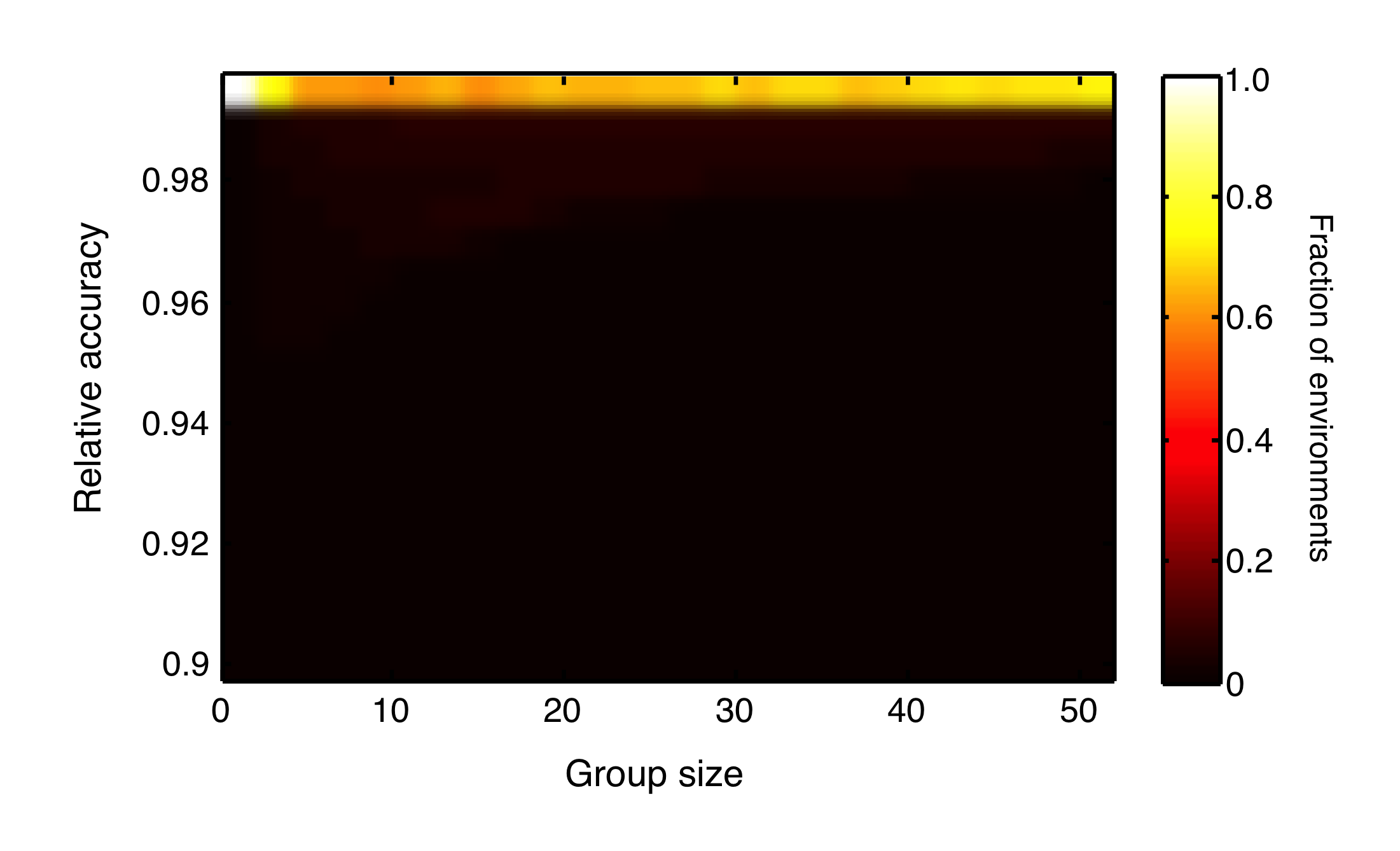

Supplement: Figure S5 — The collective accuracy resulting from the collectively learned behavior. (a–d) The mean collective accuracy for all combinations of reliabilities of the two cues, for (a) group size , (b) , (c) , and (d) . For each environment and group size combination, 500 simulations of 1000 training trials were performed, using a learning rate of , and the mean behavior of the last 100 trials across the simulations was used. (TIFF) [file pcbi.1003762.s005.tiff]

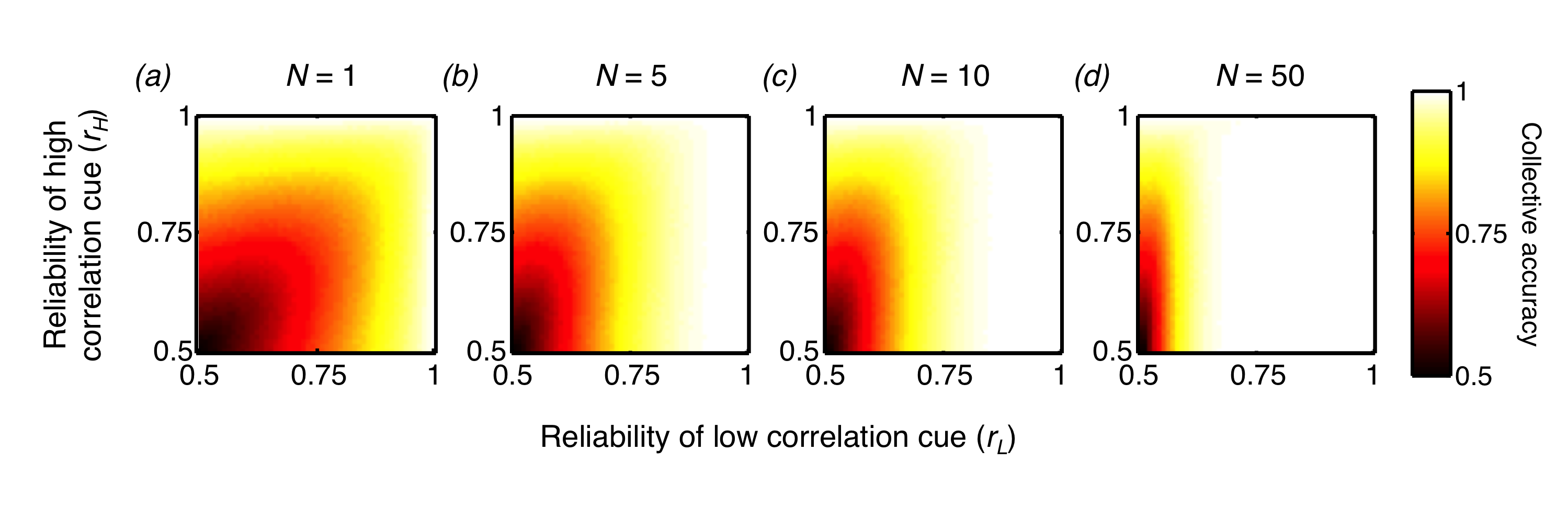

Supplement: Figure S6 — Comparison of collective accuracy resulting from the optimal voting rule with the constraint of simple majority rule to the accuracy attained when any group decision rule can be employed (the ‘global’ optimal rule). For each group size, we tested all combinations of cue reliabilities and and calculated the fraction of the accuracy of the globally optimal rule that the simple majority optimal rule achieves. Across all group sizes and environments, the simple majority optimal rule nearly always achieves greater than 99% of the accuracy of the globally optimal rule. (TIFF) [file pcbi.1003762.s006.tiff]

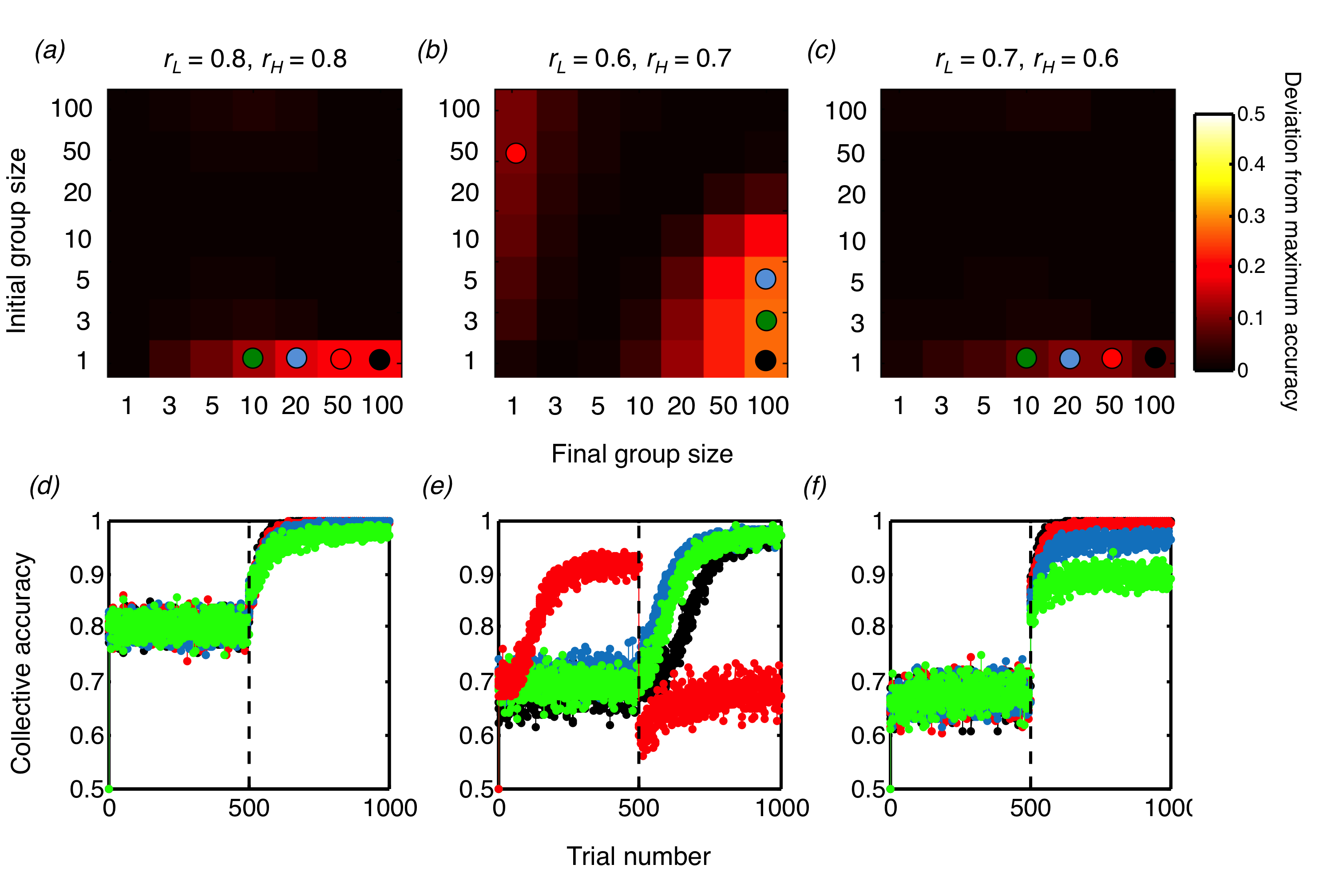

Supplement: Figure S7 — Collective learning subsequent to abrupt changes in group size for three representative environments. (a–c) We assume that individuals use the voting behavior that is optimal for the environment and starting group size (y-axis) and calculate the difference in collective accuracy that results from using that behavior in a range of new group sizes (x-axis) relative to the optimal behavior for the new group size. (d–f) We select four of the most challenging conditions in each environment (colored dots in a–c) and simulate collective learning in those contexts. Colors of lines match the dots in (a–c). Following the change in group size (which occurs after 500 trials), individuals in all conditions asymptote at close to the maximum possible for the new context. (TIFF) [file pcbi.1003762.s007.tiff]

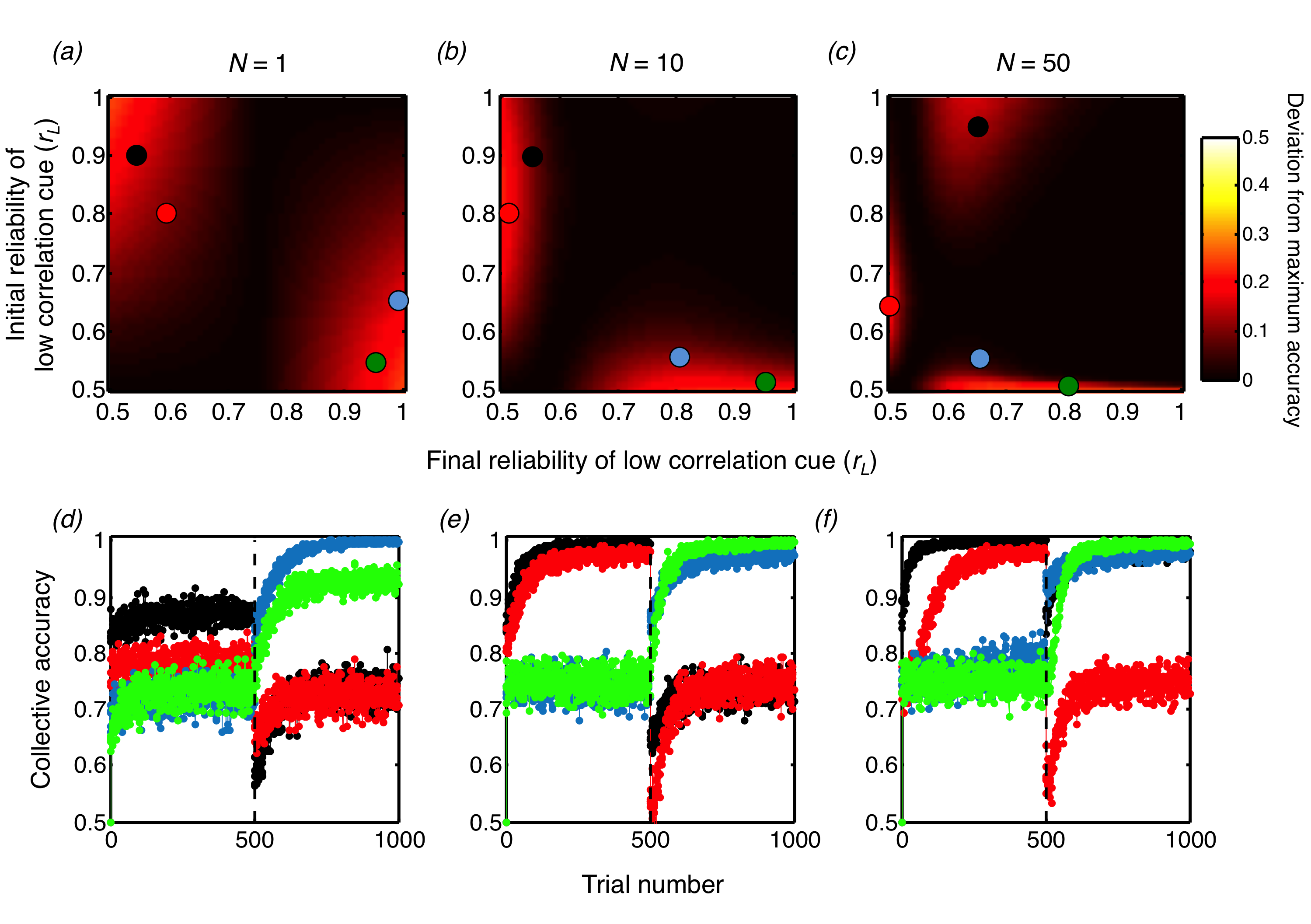

Supplement: Figure S8 — Collective learning subsequent to abrupt changes in the reliability of environmental cues for three representative group sizes. (a–c) For simplicity, we fix the reliability of the high correlation cue at and consider all combinations of changes in the reliability of the low correlation cue. We assume that individuals use the voting behavior that is optimal for the starting environment and group size (y-axis) and calculate the difference in collective accuracy that results from using that behavior in a range of ending reliabilities of the low correlation cue (x-axis) compared to the optimal behavior for that environment. (d–f) We select four of the most challenging conditions in each group size (dots in a–c) and simulate collective learning in those contexts. Colors of lines match the dots in (a–c). Following the change in cue reliability (which occurs after 500 trials), individuals in all conditions asymptote to close to the maximum possible for the new context. (TIFF) [file pcbi.1003762.s008.tiff]
