# Supplementary material for: Collective Learning and Optimal Consensus Decisions in Social Animal Groups
Source: PLoS Comput Biol. 2014 Aug 7;10(8):e1003762. doi: 10.1371/journal.pcbi.1003762 (PMC4125046; doi:10.1371/journal.pcbi.1003762)
Supplement: Text S1 — Comparing simple majority rule to a full spatial model of collective decision-making. Description of the spatial schooling model and comparison of its behavior to our assumptions of consensus and simple majority rule. (PDF) [file pcbi.1003762.s009.pdf]

Supplemental Text S1: Comparing simple majority rule to a full spatial model of collective decision-making

In our model of collective learning, we assume that groups employ simple majority rule in order to determine the collective decision. This approach was chosen for simplicity as the learning dynamics only depend on the final group consensus and not the internal decision making process. In addition to its simplicity, however, simple majority rule accurately models the outcomes of both experiments on decision-making in animal groups and much more complex decision-making models. To illustrate this point we simulated the full complexity of a spatial swarming model like those employed in [23,43]. Although there is the potential for far more complex network structures that include long-range (or topological) interactions [38,40], and explicit homophilic or heterophilic associations [44], studies show qualitatively equivalent results in a broad class of models [23,45]. These findings suggest we may expect the precise relationship between the probability of selecting the majority option, the size of the majority and the size of the group, to vary depending on model details, however the observation that even large groups are sensitive to small differences in collective preference appears robust.

In the schooling model, group consensus (here cohesive motion towards one of two targets) emerges spontaneously from noisy local interactions among individuals explicitly moving through space. The model includes both social and goal-directed behavior. Additionally, the full spatial model includes several empirically derived, yet generic, biological details, such as the primacy of avoiding collisions or a limited turning rate.

Implementation of the spatial model

Groups of N individuals are composed of two subpopulations N_A (individuals voting or with a preference for target A) and N_B (individuals voting for target B). The state of each individual at time t can be completely described by its position $\vec{c}^{\,i}(t)$, a unit velocity/heading vector $\hat{v}^{i}(t)$ and its preferred target. At each time step individuals update their positions and velocities according to both interactions with other individuals within two circular zones surrounding the individual (see supplemental figure S1) and a desire to move towards their preferred target using the following algorithm.

Spatial model algorithm

For each individual $i \in \{1, \cdots, N\}$, at each time step, do:

1a) If any other individuals are within the smaller interaction zone $(r < \varphi)$ of the focal individual, the focal individual attempts to move away in order to avoid collisions and maintain a region of personal space. This repulsion takes precedence over all other interactions.

$$\vec{s}^{\,i}(t + \Delta t) = -\sum_{j} \frac{\vec{c}^{\,j}(t) - \vec{c}^{\,i}(t)}{\left\| \vec{c}^{\,j}(t) - \vec{c}^{\,i}(t) \right\|}$$

where $\vec{s}^{\,i}(t + \Delta t)$ is the social portion of individual i's desired direction of motion and j iterates over all neighbors within this interaction zone.

1b) If the small interaction zone is empty, the focal individual is attracted toward and attempts to align with neighbors in the outer interaction zone $(\varphi < r < \vartheta)$.

$$\vec{s}^{\,i}(t + \Delta t) = \sum_{k} \frac{\vec{c}^{\,k}(t) - \vec{c}^{\,i}(t)}{\left\| \vec{c}^{\,k}(t) - \vec{c}^{\,i}(t) \right\|} + \sum_{k} \hat{v}^{k}(t) + \hat{v}^{i}(t)$$

where k is the set of all neighbors in the outer interaction zone.

2) After computing a socially desired direction of travel, each individual also incorporates their goal-oriented desired direction, weighted by a term ω which parameterizes the relative strength of goal-oriented to social-oriented influence. $\omega = 0$ indicates no goal-oriented behavior, $\omega = 1$ weights social and goal oriented behavior equally, and $\omega = \infty$ indicates no social influence. Here we use $\omega = 0.1$ such that social interactions dominate and consensus is readily achieved. $\hat{g}^{i}(t)$ indicates a unit vector pointing from the focal individual towards its desired target.

$$\vec{d}^{\,i}(t + \Delta t) = \frac{\vec{s}^{\,i}(t + \Delta t)}{\left\| \vec{s}^{\,i}(t + \Delta t) \right\|} + \omega \hat{g}^{i}(t)$$

3) The complete desired of direction travel $\vec{d}^{\,i}(t + \Delta t)$ is then normalized.

$$\hat{d}^i(t+\Delta t) = \frac{\vec{d}^i(t+\Delta t)}{\left\|\vec{d}^i(t+\Delta t)\right\|}$$

4) To model random noise and fluctuations, $\hat{d}^i(t+\Delta t)$ is rotated by ξ^i radians, where ξ^i is drawn from a circular-wrapped normal distribution (mean = 0, σ = 0.01).

5) Lastly we assume that individuals cannot change direction by more than θ radians in a given Δt. If the difference between $\hat{v}^i(t)$ and $\hat{d}^i(t+\Delta t)$ is greater than θ, $\hat{v}^i(t+\Delta t)$ is $\hat{v}^i(t)$ rotated θ towards $\hat{d}^i(t+\Delta t)$. Otherwise $\hat{v}^i(t+\Delta t) = \hat{d}^i(t+\Delta t)$. Position is then updated according to the typical kinematics.

$$\vec{c}^{\,i}(t+\Delta t) = \vec{c}^{\,i}(t) + \hat{v}^i(t+\Delta t)\Delta t$$

Following [23] we chose the following parameters: $\varphi = 1$ body length, $\vartheta = 6$ body lengths, $\omega = 0.1$, $\Delta t = 0.1$, target A location = (100, 50) (units are in body lengths), target B location = (100, -50), target radius = 10 body lengths, $\theta = 115$ degrees, $\sigma = 0.01$. Individuals begin at random positions within a circle of radius 2 centered at (0,0).

Results of the spatial model

This spatially explicit schooling model can be interpreted as an opinion dynamics model with a time-varying interaction network. We compare the degree of consensus found in this common model of collective decision-making, as well as the degree to which the collective decision follows majority rule.

To measure consensus, we simulate groups where half of the group prefers target A, and the other half prefers target B, and we observe what fraction of the group ends up deciding on each option. A high degree of consensus implies that all or nearly all of the group decides on just one of the options, whereas a low degree of consensus implies that groups split often and individuals tend to choose their preferred target. We simulated 10000 decision-making bouts

and find that the spatial schooling model consistently shows a high degree of consensus (supplemental figure S2 a-c), where in nearly all trials, the entire group selects one of the targets. This is true even in relatively large group sizes (supplemental figure S2 c), where individuals interact with only a small subset of the entire group.

Simple majority rule implies that the probability that the group will select that one of the targets is 0 when less than half of the group prefers the target, and 1 when more than half of the group prefers the target. For each group size N, we simulated 1000 decision-making bouts each of N_A individuals preferring target A and $N - N_A$ individuals preferring target B, and including all N_A between 0 and N. We then calculate the proportion of bouts in which target A was reached by the group as a function of N_A. We find that the spatial schooling model is closely approximated by simple majority rule, i.e., there is a low probability that the group decides on the minority preference (supplemental figure S2 d-f).

We focused here on the spatial model because it has been shown to accurately describe the behavior of animal groups as diverse as fish schools [23] and pigeon flocks [25]. It is also a relatively complex opinion dynamics model, in which the interaction network is time-varying, individuals are subjected to noise in their direction of travel, and individuals interact only with local neighbors. Nonetheless, we find close agreement with the two primary assumptions made in our model; individuals in groups readily make consensus decisions, and the consensus decision favors the option preferred by the majority of the group.
