# Supplementary material for: Collective Learning and Optimal Consensus Decisions in Social Animal Groups
Source: PLoS Comput Biol. 2014 Aug 7;10(8):e1003762. doi: 10.1371/journal.pcbi.1003762 (PMC4125046; doi:10.1371/journal.pcbi.1003762)
Supplement: Text S2 — Relaxing the assumption of linear voting behavior. Comparison of the collective accuracy resulting from a family of logistic (non-linear) voting behavior rules. (PDF) [file pcbi.1003762.s010.pdf]

__**Supplemental Text S2: Relaxing the assumption of linear voting behavior**__

An individual's voting behavior maps observations of the environment and learned knowledge of environmental cue (encoded as associative strengths, $V^i(t)$) to a discrete vote for one of the two options. In our model, the voting behavior is a linear function, in which the probability $p^i(t)$ of voting for the option indicated by the low correlation cue is proportional to the value of the associative strength of the low correlation cue: $p^i(t) = V_L^i(t) / \left(V_L^i(t) + V_H^i(t)\right)$. This rule is standard in many associative learning models.

In general, a reasonable voting behavior $p^i(t)$ should satisfy the following requirements: it is bounded by 0 and 1, is monotonically increasing as a function of $V_L^i(t)$, is 0.5 when $V_L^i(t) = V_H^i(t)$, and is rotationally symmetric about 0.5. There are many alternative functions besides the linear rule that satisfy these requirements. To gain insight into how alternative voting rules affect the learned behavior, we replaced the linear rule with a family of logistic functions: $p^i(t) = 1/(1+\exp(-s(a^i(t)-0.5)))$, where $a^i(t)$ is proportional to $V_L^i(t)$, and $s$ controls the steepness of the function. This function satisfies the above requirements, while allowing explicit control over the steepness of the voting rule (i.e. how sensitive behavior is to changes in the value of the associative strengths). If the logistic function governing the individual voting behavior is very steep (large value of $s$), then an individual will vote for the option indicated by the low correlation cue with high probability if $V_L^i(t) > V_H^i(t)$ and vice-versa; therefore, in this regime individuals have limited ability to exhibit mixed strategies. Conversely, if the logistic function is not steep (small value of $s$), then individuals have limited ability to exclusively follow either of the cues. Here we vary the steepness from very shallow $(s = 0.1)$ to very steep $(s = 100)$ and observe what effect the steepness has on the resulting learned behavior and hence collective accuracy for the full range of environmental conditions and across group sizes. Logistic choice (sometimes referred to as 'softmax') functions have been used extensively to model individual decision-making.

For each environmental condition and group size tested, we plot the resulting collective accuracy achieved by the learned voting behavior relative to the maximum possible for that

environment and group size (supplemental figure S3). We find that the learned behavior is relatively robust to the steepness of the voting rule, even when the logistic function used for the voting rule is substantially shallower or steeper than the linear function (supplemental figure S3). The exception is for extremely shallow voting rules $(s = 0.1)$, which take much longer to reach asymptotic behavior than the 1000 trials that we simulated. Thus, while we have chosen the standard linear mapping from associative strengths to probabilities of choosing an option, we find that the learned behavior is robust to a wide range of voting rules, as long as certain conditions are satisfied.
