# Supplementary material for: Collective Learning and Optimal Consensus Decisions in Social Animal Groups
Source: PLoS Comput Biol. 2014 Aug 7;10(8):e1003762. doi: 10.1371/journal.pcbi.1003762 (PMC4125046; doi:10.1371/journal.pcbi.1003762)
Supplement: Text S3 — Relaxing the assumption of simple majority rule. Comparison of the learned behavior resulting from a family of logistic (non-linear) collective decision rules. (PDF) [file pcbi.1003762.s011.pdf]

Supplemental Text S3: Relaxing the assumption of simple majority rule

Although we find close agreement between the behavior of the spatially explicit schooling model and our assumption of simple majority rule, we explored the effect of deviations from simple majority rule on how individuals learn. Simple majority rule implies that the probability that the group will select the option preferred by a subgroup as a function of the size of that subgroup will exhibit a step function, with the transition occurring at $N/2$ (for a two-choice scenario). We replaced this step function with a logistic curve, thereby adding noise to the collective decision making process. In this modified collective decision rule, if N_A is the number of individuals that vote for option A, then the probability that the group decision is option A is $1/(1+\exp(-10(N_A/N-0.5)))$, which approximates the behavior of fish shoals in [17].

Keeping the other model parameters constant (i.e. the environment, the individual voting rule, and the learning rule), we use this modified collective decision rule and observe differences between the learned behavior as a result of this collective decision rule and the learned behavior as a result of simple majority rule. We find the learned behavior with the logistic collective decision rule (supplemental figure S4 a-d) to be very similar to the learned behavior with simple majority rule (figure 3 a-d). Furthermore, the learned behavior with the logistic collective decision rule is similar to the optimal behavior (supplemental figure S4 e-f).

Consequently, even though in our model we use simple majority rule, relaxing this assumption by allowing noise in the collective decision does not substantially alter the collectively learned behavior of individuals.
