# Supplementary material for: Collective Learning and Optimal Consensus Decisions in Social Animal Groups
Source: PLoS Comput Biol. 2014 Aug 7;10(8):e1003762. doi: 10.1371/journal.pcbi.1003762 (PMC4125046; doi:10.1371/journal.pcbi.1003762)
Supplement: Text S4 — Proof of the optimal voting behavior for animal groups. Detailed derivation of the optimal voting behavior. (PDF) [file pcbi.1003762.s012.pdf]

Supplemental Text S4: Proof of the optimal voting behavior for animal groups

To find the optimal voting behavior, we first find the probability that a majority of individuals vote for the superior option both when the high correlation cue indicates the correct option, and the probability that a majority of individuals vote for the superior option both when the high correlation cue indicates the incorrect option, given a certain voting behavior p. We then maximize the probability that the group chooses the superior option with respect to p.

The probability that a majority of individuals vote for the correct option given that the high correlation cue indicates the correct option is:

$$\text{prob}(\text{correct} \mid r_H \text{ correct}) = \sum_{k=0}^{(N-1)/2} \binom{N}{k} (pr_L + (1-p))^{N-k} (1-(pr_L + (1-p)))^k$$

$pr_L + (1-p)$ is the probability that an individual votes for the correct option (given that the high correlation cue indicates the correct option). The first term describes the case that the individual chooses to vote for the option indicated by the low correlation cue when this cue is correct, while the second term describes the case that the individual chooses to vote for the option indicated by the high correlation cue.

Taking the derivative of this expression with respect to p, we get:

$$\frac{d\ \text{prob}(\text{correct} \mid r_H \text{ correct})}{dp} =$$

$$= \sum_{k=0}^{(N-1)/2} \binom{N}{k} (N-k)(pr_L + (1-p))^{N-k-1}(r_L - 1)(1-(pr_L + (1-p)))^k +$$

$$+ \sum_{k=0}^{(N-1)/2} \binom{N}{k} (pr_L + (1-p))^{N-k} k(1-(pr_L + (1-p)))^{k-1}(1-r_L)$$

We change the indices of the first summation by 1:

$$\frac{d\ \text{prob}(\text{correct} \mid r_H \text{ correct})}{dp} =$$

$$= \sum_{k=1}^{(N+1)/2} \binom{N}{k-1} (N-k+1)(pr_L + (1-p))^{N-k}(r_L - 1)(1-(pr_L + (1-p)))^{k-1} +$$

$$+\sum_{k=0}^{(N-1)/2}\binom{N}{k}(pr_L+(1-p))^{N-k}k(1-(pr_L+(1-p)))^{k-1}(1-r_L)$$

We find that $\binom{N}{k-1}(N-k+1)$ can be combined to form:

$$\binom{N}{k-1}(N-k+1)=\frac{N!(N-k+1)}{(k-1)!(N-k+1)!}=$$

$$=\frac{N!}{(k-1)!(N-k)!}=\frac{N!k}{k!(N-k)!}=\binom{N}{k}k$$

Substituting this back into the derivative, we get:

$$\frac{d\ \text{prob}(\text{correct}\mid r_H\ \text{correct})}{dp}=$$

$$=\sum_{k=1}^{(N+1)/2}\binom{N}{k}k(pr_L+(1-p))^{N-k}(r_L-1)(1-(pr_L+(1-p)))^{k-1}+$$

$$+\sum_{k=0}^{(N-1)/2}\binom{N}{k}(pr_L+(1-p))^{N-k}k(1-(pr_L+(1-p)))^{k-1}(1-r_L)$$

The terms within the two summations are now identical to each other, except with opposite signs, so all of the terms cancel out, except for the two cases $k=0$ and $k=(N+1)/2$. The case where $k=0$ is 0, and so the derivative reduces to:

$$\frac{d\ \text{prob}(\text{correct}\mid r_H\ \text{correct})}{dp}=$$

$$=\binom{N}{(N+1)/2}\left(\frac{N+1}{2}\right)(pr_L+(1-p))^{(N-1)/2}(r_L-1)(1-(pr_L+(1-p)))^{(N-1)/2}$$

We next find the probability that a majority of individuals vote for the superior option given that the high correlation cue indicates the incorrect option, and find this to be:

$$p(\text{correct}\mid r_H\ \text{incorrect})\ =\ \sum_{k=0}^{(N-1)/2}\binom{N}{k}(pr_L)^{N-k}(1-pr_L)^{k}$$

The term pr_L is the probability that an individual votes for the correct option. It describes the case that the individual chooses to vote for the option indicated by the low correlation cue and this cue is correct, which is the only way that the individual can vote correctly in this case, since we assume here that the high correlation cue is incorrect. Taking the derivative of this

expression with respect to *p* and using the same technique as before to cancel all but one term, we arrive at:

$$\frac{d\ \text{prob}(\text{correct} \mid r_H\ \text{incorrect})}{dp} = \binom{N}{(N+1)/2}\left(\frac{N+1}{2}\right) r_L (pr_L)^{(N-1)/2}(1-pr_L)^{(N-1)/2}$$

We want to maximize the probability of choosing the superior option overall, so we sum both expressions that we found:

$$\text{prob}(r_H\ \text{correct})\ \frac{d\ \text{prob}(\text{correct} \mid r_H\ \text{correct})}{dp} + \text{prob}(r_H\ \text{incorrect})\ \frac{d\ \text{prob}(\text{correct} \mid r_H\ \text{incorrect})}{dp} = 0$$

$$r_H \binom{N}{(N+1)/2}\left(\frac{N+1}{2}\right)(pr_L + (1-p))^{(N-1)/2}(r_L - 1)(1-(pr_L + (1-p)))^{(N-1)/2} +$$

$$+(1-r_H)\binom{N}{(N+1)/2}\left(\frac{N+1}{2}\right) r_L (pr_L)^{(N-1)/2}(1-pr_L)^{(N-1)/2} = 0$$

We then solve this expression for p using the substitutions $\alpha = \frac{1-r_H}{r_H}$, $\beta = \frac{1-r_L}{r_L}$, and $\gamma = \frac{2}{N-1}$ to simplify the final expression:

$$r_H \binom{N}{(N+1)/2}\left(\frac{N+1}{2}\right)(pr_L + (1-p))^{1/\gamma}(r_L - 1)(1-(pr_L + (1-p)))^{1/\gamma} +$$

$$+(1-r_H)\binom{N}{(N+1)/2}\left(\frac{N+1}{2}\right) r_L (pr_L)^{1/\gamma}(1-pr_L)^{1/\gamma} = 0$$

$$r_H (pr_L + (1-p))^{1/\gamma}(r_L - 1)(1-(pr_L + (1-p)))^{1/\gamma} +$$

$$+(1-r_H) r_L (pr_L)^{1/\gamma}(1-pr_L)^{1/\gamma} = 0$$

$$\frac{(pr_L)^{1/\gamma}(1-pr_L)^{1/\gamma}}{(pr_L + (1-p))^{1/\gamma}(1-(pr_L + (1-p)))^{1/\gamma}} = \frac{\beta}{\alpha}$$

$$p = \frac{(\beta^{\gamma+1} - \alpha^{\gamma})(1+\beta)}{\beta^{\gamma+2} - \alpha^{\gamma}}$$
