# Supplementary material for: Collective Learning and Optimal Consensus Decisions in Social Animal Groups
Source: PLoS Comput Biol. 2014 Aug 7;10(8):e1003762. doi: 10.1371/journal.pcbi.1003762 (PMC4125046; doi:10.1371/journal.pcbi.1003762)
Supplement: Text S5 — Comparing the optimal restricted voting rule to the globally optimal voting rule. Comparison of the individual-level optimal voting rule to the globally optimal voting rule reveals little loss of collective accuracy. (PDF) [file pcbi.1003762.s013.pdf]

Supplemental Text S5: Comparing the optimal restricted voting rule to the globally optimal voting rule

In our model of collective decision-making, individuals are free to use cues in any way to decide on which option to vote for, but the group's votes are aggregated through simple majority rule (although we showed in supplemental text S3 that the results do not depend strongly on this assumption) because simple majority rule approximates the decision-making process of many animal groups (see supplemental text S1). However, the most general voting rule should allow for any use of cues, as we do, but also any aggregation rule to combine the individual votes into a consensus group decision. We may expect this extra degree of freedom to allow the globally optimal voting rule to achieve a greater collective accuracy for a given environment than the individual-level optimal voting rule that we developed, in which votes are aggregated through simple majority rule.

We directly compare the accuracy of the individual-level optimal voting rule to the accuracy of the globally optimal voting rule for all environmental conditions and a range of group sizes and find that the decrease in collective accuracy when groups are restricted to a simple majority rule is surprisingly minimal (supplemental figure S6). Across all environmental conditions and group sizes, the restricted voting rule achieves at least 95% of the accuracy of the globally optimal voting rule, and frequently above 99% (supplemental figure S6). This demonstrates that being restricted to a simple majority rule poses little constraint on the collective accuracy that these groups could theoretically attain and supports idea that animal groups may be able to achieve a high degree of collective intelligence even under considerable cognitive and communication constraints.
